# Exploring the Effects of Early Trauma in a Forensic High Secure Population: Evaluating Associations Between Adverse Childhood Experiences (ACEs) and Diagnosis of Antisocial Personality Disorder (ASPD)

**DOI:** 10.1192/bjo.2022.216

**Published:** 2022-06-20

**Authors:** Emma McPhail, Neil Meggison, Ian Yanson, Pallab Majumder, Christian Sales

**Affiliations:** 1 Nottinghamshire Healthcare NHS Foundation Trust, Nottingham, United Kingdom; 2 Lancashire and South Cumbria NHS FT, Preston, United Kingdom; 3 Institute of Mental Health, Nottingham, United Kingdom

## Abstract

**Aims:**

To examine links between Adverse Childhood Experiences (ACE) categories and diagnosis of antisocial personality disorder (ASPD) in this population; it is predicted that there will be a positive association between number of ACEs and ASPD. The effectiveness of high secure hospital admission and treatment in reducing number of risk incidents was also examined. ACEs are known to impact significantly on the development of the personality and future psychiatric risk. Currently, research into links between distinct ACE categories and the diagnosis of ASPD in the high-secure inpatient population is limited.

**Methods:**

Data were collected from a sample (n = 221) including all patients in the Mental Health, Personality Disorder and Women's Services at a high-secure hospital. Records were examined for evidence of abuse/neglect during childhood, and a number of markers of household dysfunction. The statistical relationship between each ACE category and subsequent diagnosis of ASPD was examined through paired t-tests. Frequency of incident reports (IR1s) involving violence was compared in the first, third and fifth years post-admission.

**Results:**

Significant associations with adult diagnosis of ASPD were seen in categories of childhood physical abuse, sexual abuse, divorced/separated parents, Looked After Child (LAC) status and parental substance misuse, and total number of ACE categories present overall. Significant reductions in frequency of IR1s were seen in all services between first- and fifth- year post admission.

**Conclusion:**

A significant association between ACEs in specific domains and ASPD in adulthood was found. The importance of detailed exploration of childhood circumstances in this group is highlighted, as well as the need for further investigation of the psychological and social mechanisms underlying.

